# Determinants of stillbirths in Ghana: does quality of antenatal care matter?

**DOI:** 10.1186/s12884-016-0925-9

**Published:** 2016-06-02

**Authors:** Patience A. Afulani

**Affiliations:** Department of Community Health Sciences, Fielding School of Public Health, University of California, Los Angeles, USA; California Center for Population Research, Los Angeles, USA

**Keywords:** Antenatal care, Prenatal care, Quality of care, Stillbirths, Birth outcomes, Ghana, Sub-Saharan Africa

## Abstract

**Background:**

Each year, over two million babies die before they are born. Like maternal deaths, the great majority of these stillbirths occur in developing countries, with about a third of all cases worldwide in sub-Saharan Africa (SSA). Few studies have, however, examined the determinants of stillbirths in SSA. In addition, the emphases on promoting deliveries by skilled birth attendants and/or in health facilities to prevent maternal deaths, though important, may have undermined efforts to provide good quality antenatal care (ANC), which may have an additional role in preventing stillbirths. This study examines the factors associated with stillbirths in Ghana, focusing on the role of ANC quality.

**Methods:**

Data are from the Ghana Maternal Health Survey (*N* = 4,868)—a national survey of women of reproductive age. The main analysis includes women who had a pregnancy ending in a live birth or stillbirth in the five years preceding the survey and who received ANC at least once. ANC quality is measured by an index based on receipt (or otherwise) of nine antenatal services during the last pregnancy, including education about pregnancy complications; with receipt of at least of eight services classified as higher quality ANC. Stillbirths refer to babies born dead at seven or more months of pregnancy. Analytic techniques include multilevel logistic regression, with moderation and mediation analysis to examine conditional and intervening effects respectively.

**Results:**

Higher quality ANC decreases the odds of a stillbirth by almost half after accounting for other factors, including the type of delivery provider and facility. Educating pregnant women about pregnancy complications contributes significantly to this difference by ANC quality. The type of delivery facility and provider account for a small proportion (14 %) of the ANC quality effect on stillbirths and a larger proportion of the rural/urban difference (27 %) in stillbirths. Completing the recommended four antenatal visits decreases the odds of a stillbirth. Having a pregnancy complication, a multiple gestation, a past stillbirth, or a sister who died from pregnancy complications increases the odds of a stillbirth.

**Conclusions:**

Good quality ANC can improve birth outcomes in two ways: directly through preventative measures, and indirectly through promoting deliveries in health facilities where complications can be better managed. Targeted programs and policies to increase ANC quality, including adequately educating women on pregnancy complications, will help improve birth outcomes in Ghana, and in SSA as a whole.

**Electronic supplementary material:**

The online version of this article (doi:10.1186/s12884-016-0925-9) contains supplementary material, which is available to authorized users.

## Background

Every year, over two million babies die before they are born—an estimated 18.9 stillbirths per 1000 births globally [1]. Like maternal deaths, over 98 % of stillbirths occur in low- and middle-income countries. Sub-Saharan Africa (SSA) has the highest stillbirth rate globally—28.3 per 1000 births, compared to 3.1 for high income countries—and accounts for about a third (35.4 %) of the global burden of stillbirths [1]. Stillbirths are described as an “invisible problem” and a “hidden loss,” as they are usually not counted in local data collection systems nor considered in national and global policy and program priorities [2, 3]. Recent estimates however show that stillbirths are a huge burden to countries and health systems, especially in developing settings. The estimated numbers of stillbirths are said to be greater than that for many other conditions high on the global agenda, including HIV/AIDS; intrapartum stillbirths alone exceed global child deaths due to malaria [2, 4]. Stillbirths are also a huge burden to women and their families. The grief experienced by women with stillbirths is very high, and depression felt by mothers and families when a stillbirth occurs may exceed that associated with a neonatal death [2, 5]. Social taboos, especially in developing settings, may however prevent women from openly grieving the loss of a fetus [2].

The World Health Organization (WHO) defines a stillbirth as a fetal death (death prior to the complete expulsion or extraction of a product of conception from its mother) in the third trimester (≥28 completed weeks of gestation or birth weight ≥1000 g), for international comparisons. However, all fetal deaths after the period of viability, which may be as low as 18 weeks in some high-income countries, are considered stillbirths [1, 3]. The factors associated with stillbirths greatly overlap with those for maternal and neonatal deaths [2, 6, 7]. Hypertensive disorders, hemorrhage, anemia, malaria, and other maternal infections—risk factors for maternal deaths—are risk factors for both antepartum stillbirths (stillbirths that occur before the onset of labor, usually more than 12 hours prior to delivery; also called macerated stillbirths) and intrapartum stillbirths (stillbirths that occur after the onset of labor, usually less than 12 hours prior to delivery; also called fresh stillbirths). Prolonged or obstructed labor is the major cause of intrapartum stillbirths [2, 7, 8]. Fetal causes like congenital malformations, fetal growth restriction, prematurity, and fetal asphyxia are also all related to maternal risk factors, although the cause of stillbirths may be unknown in up to about one-third of cases [2]. Antepartum stillbirths reflect quality of antenatal care, while intrapartum stillbirths reflect quality of delivery care [2, 9–12].

Like in many countries in SSA, stillbirths are not routinely and adequately recorded and monitored in Ghana [1]. Estimates of stillbirth rates from different surveys in Ghana range from about 14 to 22 per 1000 births [13–15], with higher rates from demographic surveillance and health facility data in different parts of the country—e.g., 23 stillbirths ⁄1000 births for the Navrongo area in the Upper East region [16], 32 stillbirths/1000 births for a rural district in the Brong Ahafo region [17], to 59 stillbirths/1000 vaginal deliveries in a tertiary health facility [18]. Very few studies have examined factors associated with birth outcomes in Ghana, and none were based on national data [16, 17, 19]. Moreover, while these studies speculate that quality of maternal health services may be contributing to differential birth outcomes, most do not explicitly examine these effects. The lack of national level studies on the determinants of birth outcomes and the role of maternal health services may have occurred because the Demographic and Health Surveys (DHS) and the UNICEF multiple indicator cluster surveys—the major sources of national maternal health data for Ghana—do not collect maternal health service utilization data for pregnancies that did not result in a live birth. This study takes advantage of the Ghana Maternal Health survey (GMHS), which had a special focus on reproductive health and so collected maternal health service utilization data for all women who had a birth (live birth or stillbirth) in the five years preceding the survey.

The purpose of this study is to examine the factors associated with stillbirths in Ghana. I examine the effect of maternal risk factors, including biological and social factors, and the role of maternal health service (antenatal and delivery care) utilization and quality. In addition, the emphases on delivering with a skilled birth attendants (SBA) or in a health facility, though crucial to prevent maternal and fetal deaths, may have undermined efforts to provide good quality antenatal care (ANC), as most efforts are concentrated on increasing coverage for health facility deliveries. A major goal of this analysis is therefore to examine if quality of ANC has an effect on stillbirths net of maternal risk factors and the delivery provider or place. The measure of ANC quality is a process measure. Though good process does not necessarily result in good outcomes, this is usually the expectation [20]. Thus, while there are other factors that can influence the outcome of a pregnancy, we expect that all things being equal, good quality care during pregnancy and delivery should increase the chances of having a live baby. I therefore hypothesize that higher quality ANC will be associated with lower risk of delivering a stillbirth net of other factors.

## Methods

### Data

The data for this analysis are from the 2007 Ghana Maternal Health Survey. The GMHS was the first (and remains the only) nationally representative population-based survey to collect comprehensive information on maternal morbidity and mortality in Ghana. The survey was conducted by the Ghana Statistical Service and the Ghana Health Service with technical assistance from Macro International, and has been described in detail elsewhere [13, 21]. Based on a multistage cluster design, households were randomly selected from all regions of Ghana and administered household and women’s questionnaires. The response rate was 99 % at the household level and 98 % for the individual women, with 10,858 completed household interviews and 10,370 individual interviews with women aged 15–49 years. Only women who had a birth (live or still birth) in the five years preceding the survey were asked the questions on maternal health services and birth outcomes (*N* = 5,088 = 49 % of all women interviewed); this is thus the base sample for the analysis. The analytic sample is however 5,042 women (99.1 % of the base sample) because 46 observations are missing on key study variables (including 34 missing antenatal attendance and 18 missing delivery attendant). The multivariate analysis is further restricted to women who had at least one ANC visit during their last pregnancy, since quality of ANC cannot be measured for women who did not have any ANC—97 % (*N* = 4,868) of women in analytic sample had at least one antenatal visit. The full analytic sample is used in sensitivity analysis.

Ethical approval for the survey was obtained from the Ghana Health Service Ethical Review Committee and verbal consent was obtained from respondents. This study was granted an exemption under the University of California, Los Angeles Institutional Review Board exemption category 4 for research involving the study of existing data.

### Constructs and variables

#### Dependent variable: birth outcome

Birth outcome in this study refers to whether a woman had a stillbirth or a live birth in her last pregnancy. Babies reported as born dead—baby did not cry, move, or breathe when it was born—and of pregnancy duration seven months or above were classified as stillbirths in the survey. Because the questions on use of maternal health services were only asked of those who had a live birth or stillbirth, pregnancies that ended before seven months (miscarriages or induced abortions) are not included in this analysis. Birth outcome is therefore a binary variable coded ‘1’ for stillbirths and ‘0’ for live births.

#### Independent variables

*Quality of antenatal care* is the key independent variable in the analysis, and I define high quality care as receiving the ANC services recommended by WHO and the Ghana Health Service during pregnancy [20, 22, 23]. ANC quality is calculated as an additive index based on responses to nine questions about whether or not women received nine services during any of their ANC visits for their last pregnancy. The services are: being weighed, blood pressure checked, a urine sample taken, a blood sample taken, education received on signs of pregnancy complications, education received on where to go in the event of a complication, received or told to buy iron supplements, received an anthelminthic, and received tetanus vaccination. Each question has a binary response (1 = Yes; and 0 = No), so the index ranges from zero to nine with responses spanning the entire range. Details on the construction of this index have been previously described [21]. For this analysis, the index is used as a dichotomous variable—lower quality = received zero to seven services (coded ‘0’); and higher quality = received eight or nine services (coded ‘1’).

##### Health service utilization

These include (1) *antenatal services*: frequency of ANC visits (less than four or the recommended four plus visits), trimester of the first ANC visit (first, second, or third trimester), type of ANC provider (doctors, nurse/midwife, or other) and type and level of ANC facility (government hospital/polyclinic, government health center/health post/other lower tiered health facility, private clinic/maternity home, or not a health facility); and (2) *delivery services*: type of delivery provider (examined in two ways: delivery by a SBA or not; and delivery by doctors, nurse/midwife, and others), and place of delivery (examined in two ways: delivery in a health facility or not; and delivery in a government hospital/polyclinic, a government health center/health post/other lower tiered health facility, a private clinic/maternity home, or not a health facility).

##### Maternal risk factors for adverse birth outcomes

The following were examined based on the literature on the determinants of stillbirths [2, 16, 17, 19, 24]: Age, gravidity (number of pregnancies), a complication during the index pregnancy (i.e., reported signs or symptoms of hemorrhage, preeclampsia/eclampsia, infection, obstructed labor, etc.), multiple gestation (i.e., not a singleton) in the index pregnancy, past stillbirth, past miscarriage, and past induced abortion. Having a sister who died from pregnancy complications was found to be an important stillbirth determinant in preliminary analysis, hence is included here. In addition, two variables to capture other unmeasured maternal conditions that increase the risk of having a still birth were included: the reason for ANC (for a problem or check up) and the receipt of any intervention during delivery (caesarian delivery, forceps delivery, receipt of blood transfusion, and receipt of intravenous (IV) fluids; these are correlated and so were combined to create a binary variable coded: 1–receipt of any intervention; and 0–no intervention).

##### Sociodemographic factors

I also investigated social determinants of birth outcomes, use of health services, and quality of care, both because they are important and also reduce endogeneity in the key relationships. These variables include place of residence (rural/urban residence and region of residence), socioeconomic status (education and wealth), religion, ethnicity, marital status, age at first union, and familiarity with the health system (using knowledge of where to get contraception and use of contraception as proxies). These variables are distal determinants that could potentially affect birth outcomes through their effect on utilization and/or quality of maternal health services.

### Statistical analysis

Initial analyses included descriptive statistics—means for continuous variables and proportions for categorical variables—and bivariate associations between the independent and the dependent variables. Chi-squared tests were used to assess significant differences in birth outcome [25, 26]. The descriptive statistics and cross tabulations are all weighted using the sample weights provided with the data to account for the complex sampling design. To account for the hierarchical nature of the data, I estimated bivariate and multivariate multilevel logistic regression models. Multilevel regression is necessary because clustering at various levels results in underestimation of the standard errors and potentially biased coefficients [27, 28]. Two levels are included in this analysis: level 1 is the individual and level 2 is the survey cluster (There are 400 clusters; average number of observations per cluster is 12; minimum of 3 and maximum of 38 observations per cluster. Preliminary analysis showed that only the variation between individuals and clusters was significant for birth outcomes, and a two level multilevel regression was preferred to a single level logistic regression). The weights provided with the datasets are only for the individual level and there is not enough information to construct weights for use in the multilevel analysis. Furthermore, there is no clear consensus on the use of weights in multilevel analysis within the field of statistics [29]. I therefore estimated unweighted multilevel models. The “xtmelogit” command in Stata was used to estimate the multilevel binary logistic regression models with random intercepts [28, 30].

The final multivariate model includes only the variables that are statistically significant in the bivariate models and those for which there is strong empirical or theoretical rationale for their inclusion. Some variables are, however, excluded from the final multivariate model because of multicollinearity.

I also examine for intervening and conditional effects: (1) if the delivery provider/place mediates the effect of ANC quality on the birth outcome; (2) if ANC quality or the delivery provider/place mediate the effects of social factors like place of residence and socioeconomic status (SES) on the birth outcome; and (3) if delivery provider/place, place of residence, or SES moderates the effect of ANC quality on the birth outcome. For the mediation analysis, because the addition of variables to a logistic model changes its scale, it is not accurate to consider the difference in the coefficients in nested logistic models as the mediated effect itself [31, 32]. I therefore used the ‘khb’ rescaling method which applies the residual of the potential mediators to the reduced model to fix the scale of the reduced model to that of the full model, so that the coefficient for the key independent variable can be compared across the nested models [31, 33]. The conditional effects were examined by including interactions terms for quality of ANC and: the delivery provider, delivery facility, rural/urban residence, education, and wealth.

## Results

### Sample distribution

Table 1 shows the distribution of the study variables for women who received ANC at least once for the weighted and unweighted samples, which are similar. The weighted distribution is described here unless otherwise specified. Among women who had at least one ANC visit 1.5 % (77 out of 4,868) had a stillbirth, giving a crude stillbirth rate of about 15 per 1000 births. The Crude birth rate for the full sample is about 17 per 1000 births (85 out of 5042), shown in Additional file 1; the distributions for the full sample are not significantly different from that for the restricted sample described here. Over two thirds of the stillbirths occurred in the ninth month of pregnancy. The average woman in the sample is about thirty years old. Most women are currently married (72 %) and 14 % are cohabiting. On average, the women have had about four pregnancies. About 4 % had a past stillbirth; 20 % had some pregnancy complication during their last pregnancy; 3 % had a multiple pregnancy; and about 2 % had a sister who died from pregnancy complications. On average, women had about 5 years of education; about a third have no formal education. About two-thirds of the women live in rural areas; and all regions of Ghana are represented in the sample.

**Table 1:** Sample distribution, Women who attended antenatal care (ANC) at least once, Ghana Maternal Health Survey (GMHS), 2007 (*N* = 4,868)

	*Unweighted*		*Weighted*	
*Variables*	N	%	%	[95 % C.I]
Last Birth outcome				
Live birth	4,791	98.4	98.5	[98.1 98.9]
Stillbirth	77	1.6	1.5	[1.1 1.9]
Pregnancy duration^a^				
7 months	13	16.9	14.7	[. .]
8 months	7	9.1	9.1	[. .]
9 months	53	68.8	70.0	[. .]
10 months	4	5.2	5.4	[. .]
Age in years				
15–19 years	236	4.8	4.9	[4.2 5.6]
20–24	891	18.3	18.5	[17.1 19.8]
25–29	1,138	23.4	23.0	[21.6 24.4]
30–34	1,082	22.2	22.6	[21.3 23.8]
35–39	881	18.1	18.2	[17.1 19.4]
40–49 years	640	13.1	12.8	[11.8 13.9]
Mean	4,868	30.5	30.4	[30.2 30.7]
Marital status				
Currently married	3,510	72.1	71.8	[69.7 73.9]
Cohabiting	666	13.7	14.1	[12.5 15.7]
Previously married	347	7.1	7.0	[6.1 7.8]
Never married	345	7.1	7.1	[6.2 8.0]
No. of pregnancies (Gravidity)				
One	819	16.82	16.6	[15.4 17.8]
Two	810	16.64	16.1	[15.0 17.4]
Three	814	16.72	17.1	[16.0 18.3]
Four	692	14.22	14.5	[13.5 15.6]
Five or more	1,733	35.6	35.7	[34.1 37.4]
Mean	4,868	3.9	3.9	[3.9 4.0]
Past Stillbirth	220	4.5	4.4	[3.8 5.0]
Past miscarriage	791	16.2	15.6	[14.4 16.9]
Past induced abortion	767	15.8	15.2	[13.6 16.7]
Pregnancy complication	1,050	21.6	20.4	[19.0 21.8]
Multiple gestation	126	2.6	2.5	[2.0 3.0]
Sister maternal death	85	1.7	1.9	[1.5 2.3]
Highest Education				
None	1,588	32.6	33.0	[29.6 36.4]
Primary	1,072	22.0	22.1	[20.2 23.9]
Middle/JSS	1,804	37.1	37.5	[34.5 40.4]
Secondary/SSS/ higher	404	8.3	7.5	[6.3 8.6]
Mean years education	4,868	5.2	5.1	[4.8 5.4]
Household wealth index				
Poorest	1,024	21.0	20.7	[17.7 23.6]
Poorer	943	19.4	21.0	[18.6 23.5]
Middle	930	19.1	20.4	[18.2 22.7]
Richer	976	20.0	20.3	[18.1 22.4]
Richest	995	20.4	17.6	[15.5 19.7]
Religious affiliation				
Catholic	661	13.6	13.6	[11.5 15.8]
Methodist/Presbyterian	652	13.4	14.4	[12.7 16.1]
Pentecostal/Charismatic	1,444	29.7	28.3	[26.4 30.2]
Other Christian	810	16.6	16.8	[15.2 18.5]
Moslem	863	17.7	18.3	[15.0 21.7]
Traditional/other	438	9.0	8.5	[6.9 10.1]
Ethnicity				
Akan	2,197	45.1	47.1	[43.2 50.9]
Ga/Dangme/Guan	504	10.4	9.2	[7.1 11.2]
Ewe	615	12.6	12.0	[9.7 14.3]
Mole-Dagbani/Hausa	583	12.0	13.1	[9.6 16.6]
Grussi/Gruma	534	11.0	10.3	[7.2 13.4]
Other	435	8.9	8.4	[6.3 10.5]
Setting				
Rural	2,967	60.9	64.8	[61.7 67.9]
Urban	1,901	39.1	35.2	[32.1 38.3]
Region				
Greater Accra	619	12.7	9.5	[8.0 11.1]
Central	429	8.8	9.9	[8.3 11.6]
Western	371	7.6	8.2	[6.4 10.1]
Volta	389	8.0	9.2	[6.7 11.7]
Eastern	724	14.9	11.7	[10.3 13.1]
Ashanti	837	17.2	18.9	[16.5 21.4]
Brong Ahafo	486	10.0	11.7	[9.9 13.5]
Northern	491	10.1	13.1	[9.7 16.5]
Upper east	298	6.1	4.8	[3.7 5.8]
Upper west	224	4.6	3.0	[2.1 3.8]
No. of ANC visits				
1–3 visits	990	20.3	20.2	[18.4 22.1]
4 or more	3,878	79.7	79.8	[77.9 81.6]
Mean	4,868	5.8	5.8	[5.6 5.9]
Trimester of first ANC visit				
First trimester	2,688	55.2	54.9	[52.9 56.8]
Second trimester	1,992	40.9	41.3	[39.6 43.1]
Third trimester	181	3.7	3.6	[3.0 4.2]
Where ANC took place				
Gov’t health facility^b^	4,119	84.6	85.3	[82.9 87.7]
Gov’t hospital/polyclinic	2,200	45.2	45.3	[41.3 49.2]
Other Gov’t facility	1,919	39.4	40.0	[36.1 43.9]
Only Private facility/maternity home	703	14.4	14.0	[11.6 16.4]
Highest trained ANC provider				
Doctor	1,006	20.7	19.4	[17.6 21.3]
Nurse	3,743	76.9	78.5	[76.6 80.3]
All others	119	2.4	2.1	[1.5 2.6]
Reason for seeking ANC				
For checkup	4,044	83.1	83.1	[81.7 84.6]
For a problem	824	16.9	16.9	[15.4 18.3]
ANC quality score				
7 or less	1,901	39.1	39.1	[36.4 41.8]
8 or 9	2,967	61.0	60.9	[58.2 63.6]
Mean	4,868	7.4	7.4	[7.3 7.5]
Delivery assisted by				
SBA	2,876	59.1	57.3	[54.1 60.5]
Doctor	493	10.1	9.6	[8.4 10.7]
Nurse/Midwife	2,331	47.9	46.7	[43.7 49.7]
Auxiliary nurse/midwife	52	1.1	1.0	[0.6 1.4]
Not a SBA	1,992	40.9	42.7	[39.5 45.9]
Trained TBA	943	19.4	20.1	[17.9 22.3]
Untrained TBA	421	8.6	9.0	[7.5 10.4]
Relative/friend	473	9.7	10.5	[8.6 12.4]
No one	154	3.2	3.1	[2.5 3.7]
Other/DK	1	0.0	0.0	[0.0 0.1]
Type of Delivery facility				
Not health facility	2,029	41.7	43.7	[40.4 46.9]
Health facility	2,839	58.3	56.3	[53.1 59.6]
Type of facility^c^				
Gov’t hospital/polyclinic	1,530	53.9	53.8	[50.1 57.6]
Other Gov’t facility	689	24.3	26.2	[22.7 29.6]
Private clinic/maternity home	620	21.8	20.0	[17.2 22.8]
Delivery assisted by^c^				
Doctor	491	17.3	16.9	[15.0 18.8]
Nurse/Midwife	2,265	79.8	80.4	[78.3 82.5]
Auxiliary nurse/midwife	41	1.4	1.4	[0.8 2.0]
Trained TBA	34	1.2	1.1	[0.6 1.5]
Other	8	0.3	0.2	[−0.1 0.6]
Intervention during delivery^c^				
No	1,702	60.0	60.4	[58.0 62.8]
Yes	1,137	40.0	39.6	[37.2 42.0]
Caesarian delivery	346	12.2	12.1	[10.6 13.6]
Forceps	94	3.3	3.3	[2.5 4.1]
Blood transfusion	92	3.2	2.9	[2.2 3.5]
IV Infusion	1,016	35.8	35.1	[32.9 37.4]
Number of clusters	400			
Number of women	4,868			

Notes: The denominator for all is the total analytic sample (4,868) except for ^a^where the denominator is total stillbirths (77) and ^c^ where the denominator is deliveries in a health facility (2,839) . ^b^refers to people who had some ANC from a government facility but 98 % were exclusively in a government facility

About 80 % of women had four or more ANC visits, as recommended by WHO, with an average of about six visits; 55 % started ANC in the first trimester. About eight out of every ten women received ANC in a government health facility (85 %)—roughly half in a hospital or polyclinic and the other half in a health center or other lower-level facility—from a nurse or midwife (79 %), and for a checkup (83 %). The mean score on the ANC quality index is 7.4 and about 61 % received higher quality ANC (i.e., 8 or 9 out of the 9 services). A SBA assisted a little over half of the women at delivery—10 % by doctors and 47 % by nurses or midwives. Of the 55 % of deliveries in a health facility, 54 % were in a government hospital or polyclinic, 26 % in a government health center or health post, and 20 % in a private clinic or maternity home. Among women who delivered in a health facility about 40 % received some kind of intervention—mostly intravenous fluids (35 %); about 3 % received a blood transfusion, 3 % had a forceps delivery, and 12 % were delivered by caesarian section (7 % of all women in the sample were delivered by caesarian section).

### Bivariate results

The stillbirth rate is higher among the small group of women who did not receive any ANC—5.6 % compared to 1.5 % among women who received some ANC—but this difference is not significant, potentially due to the very small proportions, which applies to most of the other bivariate distributions of stillbirths. The bivariate results (weighted and unweighted distribution of stillbirths and unadjusted multilevel binary logistic regression results) for women who attended at least one ANC are shown in Table 2. These results are generally consistent. Among women who received some ANC, the stillbirth rate is higher among women who received lower quality ANC (1.8 %) compared to those who received higher quality ANC (1.3 %). When only clustering is accounted for, women who received higher quality ANC have about 40 % lower odds of delivering a stillbirth than those who received lower quality ANC. About 2 % of women who were assisted by a SBA and those who delivered in a health facility reported a stillbirth, compared to about 1 % for those who were not assisted by a SBA and those who delivered at home. Stillbirths are also higher for births assisted by doctors—5.1 % compared to 1.6 % for those assisted by nurses, and 1 % for others—and for births in government hospitals and polyclinics—3.1 % compared to about 1 % in other government facilities and private facilities. Having some intervention during pregnancy is positively associated with having a stillbirth.

**Table 2:** Bivariate distribution of stillbirths, Women who attended ANC at least once, GMHS (*N* = 4868)

	*Cross tabulations*				*Multilevel logistic regression*
	Unweighted		Weighted		Unweighted
*Variable*	N	%	%	[95 % C.I]	Unadjusted OR [95 % C.I]
ANC quality score					
7 or less	39	2.1	1.8	[1.2 2.5]	
8 or 9	38	1.3	1.3	[0.9 1.8]	0.62* [0.39 0.98]
No. of ANC visits					
1–3 visits	21	2.1	2.1	[1.1 3.2]	
Four or more	56	1.4	1.4	[0.9 1.8]	0.66 [0.39 1.11]
Trimester of first ANC visit					
First trimester	40	1.5	1.4	[0.9 1.9]	
Second trimester	34	1.7	1.7	[1.1 2.4]	1.14 [0.71 1.82]
Third trimester	3	1.7	0.8	[−0.1 1.8]	1.1 [0.33 3.65]
Type of ANC facility					
Gov’t hospital or polyclinic	48	2.2	1.9	[1.3 2.5]	
Other Gov’t facility	24	1.3	1.4	[0.8 2.0]	0.59* [0.35 0.98]
Private facility/maternity	5	0.7	0.7	[0.0 1.3]	0.33* [0.13 0.83]
Highest trained ANC provider					
Nurse	52	1.4	1.3	[0.9 1.8]	
Doctor	24	2.4	2.3	[1.2 3.3]	1.74* [1.05 2.89]
All others	1	0.8	0.8	[−0.8 2.3]	0.67 [0.10 5.07]
Reason for seeking ANC					
For checkup	65	1.6	1.5	[1.1 1.9]	
For a problem	12	1.5	1.5	[0.6 2.5]	0.9 [0.48 1.69]
Delivery by SBA					
No	17	0.9	0.7	[0.3 1.0]	
Yes	60	2.1	2.1	[1.5 2.8]	2.59*** [1.47 4.57]
Delivery assisted by					
Nurse/Midwife	33	1.4	1.6	[0.9 2.3]	
Doctor	27	5.5	5.1	[2.9 7.2]	4.15*** [2.43 7.11]
Other	17	0.8	0.7	[0.3 1.0]	0.56 [0.31 1.03]
Delivery in health facility					
No	18	0.9	0.7	[0.4 1.1]	
Yes	59	2.1	2.1	[1.5 2.8]	2.48** [1.43 4.33]
Type of Delivery facility					
Gov’t hospital or polyclinic	48	3.1	3.1	[2.2 4.1]	
Other Gov’t facility	6	0.9	1.0	[0.2 1.8]	0.27** [0.11 0.64]
Private clinic/maternity	5	0.8	0.9	[0.0 1.8]	0.25** [0.10 0.63]
Home/other/DK	18	0.9	0.7	[0.4 1.1]	0.26*** [0.15 0.46]
Caesarian delivery					
No	62	1.4	1.3	[0.9 1.7]	
Yes	15	4.3	4.7	[2.1 7.3]	3.34*** [1.84 6.07]
Forceps delivery					
No	68	1.4	1.4	[1.0 1.8]	
Yes	9	9.6	8.0	[2.3 13.8]	7.11*** [3.30 15.3]
Blood transfusion					
No	71	1.5	1.4	[1.0 1.8]	
Yes	6	6.5	6.3	[1.1 11.4]	4.68*** [1.90 11.5]
IV Infusion					
No	45	1.2	1.0	[0.7 1.4]	
Yes	32	3.1	3.4	[2.0 4.7]	2.75*** [1.71 4.42]
Any intervention					
No	37	1.0	0.9	[0.6 1.2]	
Yes	40	3.5	3.8	[2.5 5.1]	3.70*** [2.32 5.90]
Past Stillbirth					
No	63	1.4	1.3	[1.0 1.7]	
Yes	14	6.4	5.4	[2.5 8.4]	4.93*** [2.66 9.16]
Past miscarriage					
No	63	1.5	1.5	[1.1 1.9]	
Yes	14	1.8	1.7	[0.7 2.8]	1.13 [0.63 2.05]
Past induced abortion					
No	56	1.4	1.3	[0.9 1.7]	
Yes	21	2.7	2.5	[1.3 3.7]	1.93* [1.16 3.21]
Pregnancy complication					
No	39	1.0	0.9	[0.6 1.2]	
Yes	38	3.6	3.9	[2.4 5.3]	3.70*** [2.33 5.88]
Type of Gestation					
Single pregnancy	66	1.4	1.3	[1.0 1.7]	
Multiple pregnancy	11	8.7	8.5	[3.4 13.6]	7.45*** [3.64 15.3]
Sister maternal death					
No	70	1.5	1.4	[1.0 1.8]	
Yes	7	8.2	7.7	[1.5 13.9]	6.51*** [2.76 15.3]
Current age in years					
15–19	4	1.7	2.0	[−0.1 4.0]	1.61 [0.62 4.18]
20–24	10	1.1	1.1	[0.4 1.9]	0.91 [0.44 1.89]
25–29 (ref)	17	1.5	1.4	[0.7 2.1]	
30–34	14	1.3	1.0	[0.4 1.5]	0.82 [0.40 1.66]
35–39	13	1.5	1.7	[0.6 2.7]	0.92 [0.44 1.90]
40–49	19	3.0	2.8	[1.4 4.3]	2.16* [1.12 4.15]
Marital status					
Currently married	54	1.5	1.4	[1.0 1.9]	
Cohabitating	6	0.9	0.8	[0.1 1.4]	0.58 [0.24 1.37]
Previously married	5	1.4	1.3	[0.1 2.4]	0.95 [0.37 2.42]
Never married	12	3.5	3.9	[1.7 6.1]	2.25* [1.17 4.34]
No. of Pregnancies (Gravidity)					
One	10	1.22	1.2	[0.6 2.3]	
Two	12	1.48	1.4	[0.8 2.5]	1.22 [0.52 2.88]
Three	10	1.23	1.2	[0.6 2.1]	1.05 [0.43 2.55]
Four	8	1.16	1.1	[0.5 2.3]	0.96 [0.37 2.46]
Five or more	37	2.14	2.1	[1.4 3.0]	1.82 [0.89 3.73]
Education					
None	20	1.3	1.0	[0.4 1.5]	
Primary	13	1.2	1.4	[0.6 2.2]	0.99 [0.48 2.04]
Middle/JSS	35	1.9	1.9	[1.2 2.7]	1.56 [0.88 2.80]
Secondary/SSS/higher	9	2.2	2.2	[0.4 4.0]	1.81 [0.79 4.13]
Household wealth index					
Poorest	16	1.6	1.6	[0.9 2.8]	
Poorer/Middle	22	1.2	1.1	[0.7 1.8]	0.76 [0.39 1.48]
Rich/Richest	39	2.0	1.9	[1.3 2.7]	1.29 [0.69 2.43]
Setting					
Rural	34	1.1	1.1	[0.7 1.5]	
Urban	43	2.3	2.3	[1.5 3.1]	2.05** [1.25 3.36]
Region					
Greater Accra	9	1.5	1.0	[0.3 1.8]	
Central	7	1.6	1.3	[0.3 2.3]	1.14 [0.39 3.31]
Western	1	0.3	0.4	[−0.3 1.1]	0.19 [0.02 1.53]
Volta	3	0.8	0.8	[−0.1 1.8]	0.54 [0.14 2.13]
Eastern	17	2.3	2.6	[1.0 4.2]	1.68 [0.70 4.03]
Ashanti	15	1.8	1.7	[0.7 2.8]	1.3 [0.53 3.18]
Brong Ahafo	14	2.9	2.8	[1.2 4.5]	2.16 [0.85 5.47]
Northern	8	1.6	1.3	[0.2 2.4]	1.15 [0.40 3.27]
Upper East	2	0.7	0.7	[−0.2 1.6]	0.47 [0.09 2.33]
Upper West	1	0.4	0.4	[−0.4 1.3]	0.3 [0.04 2.55]
Religious affiliation					
Catholic	13	2.0	1.5	[0.5 2.5]	
Methodist/Presbyterian	12	1.8	1.6	[0.6 2.6]	0.78 [0.45 1.32]
Pentecostal/charismatic	21	1.5	1.7	[0.9 2.4]	0.85 [0.42 1.72]
Other Christian	13	1.6	1.6	[0.7 2.4]	0.47 [0.16 1.40]
Moslem	14	1.6	1.5	[0.6 2.3]	
Traditional/other	4	0.9	0.9	[0.0 1.8]	
Ethnicity					
Akan	36	1.6	1.6	[1.0 2.2]	
Ga/Dangme/Guan	11	2.2	2.1	[0.3 3.9]	1.3 [0.63 2.70]
Ewe	10	1.6	1.4	[0.4 2.4]	1.01 [0.48 2.13]
Mole-Dagbani/Hausa	9	1.5	1.4	[0.4 2.5]	0.89 [0.40 2.00]
Grussi/Gruma	4	0.7	0.7	[0.0 1.4]	0.45 [0.15 1.32]
Other/4missing	7	1.6	1.8	[0.3 3.2]	1.03 [0.44 2.44]
		*Random effects for null model*			
Cluster variance					0.86* [0.49 1.51]
Number of clusters	400				
Number of women	4868				

Notes: *OR* Odds Ratio. *p < 0.05, ** *p* < 0.01, *** *p* < 0.001. The first category for each variable is the reference for the logistic regression unless otherwise specified

Among the risk factors for adverse birth outcomes, having a past miscarriage and past induced abortion are associated with a slightly higher percentage of stillbirths. There is however a bigger difference by past stillbirth, with 5.4 % of those reporting a past stillbirth delivering a stillbirth compared to 1.5 % of those with no past stillbirth. Also, women who reported a complication and those with a multiple pregnancy had significantly higher stillbirths—3.9 % percent compared to 0.9 % percent for those with no complication, and 8.5 % compared to 1.3 % for singleton pregnancies, respectively. Women who reported a sister dying from a pregnancy complication also had a significantly higher proportion of stillbirths (7.7 %) than those with no such history (1.4 %). Education and wealth are not significantly associated with stillbirths, though the directions of the associations are generally positive—higher proportions of stillbirths with higher education and wealth. Stillbirths are higher in urban areas (2.3 %) than rural areas (1.3 %), with about two times higher odds of delivering a stillbirth in urban compared to rural residence.

The significant cluster variance for the null model from the multilevel logistic regression of stillbirths (at the bottom of the Table 2), which gives an approximate intra-class correlation (ICC) of 0.2, is evidence of clustering, although most of the variation is between individuals. The full multivariate model explains about 34 % of the variation between clusters.

### Multilevel multivariate binary logistic regression results

The results of the final multilevel multivariate logistic regression models for women who attended ANC at least once are shown in Table 3. Because the delivery variables are consequent to quality of ANC—i.e., come after ANC in temporal ordering—and some may have occurred after the outcome (we don’t have information on the timing of the stillbirth relative to seeking delivery care), two sets of multivariate models are presented: the first excludes all the delivery variables and the second includes them. An intervening model, which excludes whether the woman had an intervention during delivery, is not shown because the results are essentially the same as that in the model including it. Net of other factors, higher quality ANC is still significantly associated with better birth outcomes. Women who had higher quality ANC have about 50 % lower odds of delivering a stillbirth than those who received lower quality ANC—even when the delivery factors are accounted for. When other factors are accounted for, women who attended ANC four or more times have about 60 % lower odds of delivering a stillbirth compared to those who attended less than four times.

**Table 3:** Multilevel multivariate binary logistic regression of birth outcome on quality of antenatal are and relevant confounders, GMHS, *N* = 4868

	No delivery variables	Includes delivery variables
*Independent variables*	*Adjusted Odds of having a Stillbirth: OR [95 % C.I]*	
*Fixed effects*		
Higher ANC Quality	0.55* [0.33 0.92]	0.50** [0.29 0.84]
ANC Four or more times	0.49* [0.27 0.88]	0.41** [0.22 0.76]
ANC provider		
Nurse (ref)		
Doctor	1.62 [0.90 2.93]	1.43 [0.79 2.58]
All others	0.95 [0.11 8.06]	1.25 [0.14 11.5]
ANC for problem	0.81 [0.42 1.57]	0.79 [0.40 1.55]
Delivery by a SBA		2.28 [0.34 15.2]
Type of delivery facility		
Gov’t hospital or polyclinic (ref)		
Other Gov’t facility		0.36* [0.14 0.95]
Private facility/maternity home		0.26** [0.097 0.68]
Home/other/DK		0.85 [0.13 5.72]
Intervention during delivery		1.93* [1.05 3.56]
Current age in years	1.05* [1.00 1.11]	1.04 [0.99 1.10]
Number of pregnancies	0.93 [0.79 1.09]	0.96 [0.81 1.13]
Marital Status		
Currently married (ref)		
Cohabitating	0.79 [0.31 1.98]	0.92 [0.36 2.31]
Previously married	0.89 [0.34 2.37]	0.93 [0.35 2.48]
Never married	3.52** [1.58 7.85]	3.13** [1.39 7.09]
Past Stillbirth	3.71*** [1.83 7.53]	3.46*** [1.67 7.14]
Past abortion	1.63 [0.88 3.00]	1.58 [0.85 2.93]
Pregnancy complication	3.10*** [1.89 5.08]	2.68*** [1.61 4.45]
Multiple gestation	6.26*** [2.84 13.8]	4.93*** [2.21 11.0]
Sister maternal death	5.69*** [2.19 14.8]	5.58*** [2.09 14.9]
Years of sch. centered	1.04 [0.98 1.11]	1.03 [0.96 1.10]
Household wealth Index		
Poorest (ref)		
Poorer/Middle	0.61 [0.29 1.29]	0.57 [0.27 1.21]
Rich/Richest	0.71 [0.28 1.80]	0.55 [0.21 1.43]
Urban residence:	2.09* [1.03 4.25]	1.79 [0.86 3.72]
Region		
Greater Accra (ref)		
Central	2.38 [0.76 7.40]	2.57 [0.81 8.18]
Western	0.38 [0.045 3.23]	0.42 [0.049 3.64]
Volta	1.33 [0.31 5.73]	1.38 [0.32 6.04]
Eastern	2.64* [1.02 6.84]	2.72* [1.05 7.07]
Ashanti	1.72 [0.68 4.37]	1.6 [0.63 4.11]
Brong Ahafo	4.17** [1.48 11.8]	4.69** [1.65 13.3]
Northern	2.83 [0.88 9.16]	3.33* [1.01 11.0]
Upper east	1.86 [0.32 11.0]	1.91 [0.32 11.5]
Upper west	0.98 [0.11 9.04]	0.96 [0.10 8.95]
Constant	0.0015*** [0.00026 0.0091]	0.0018*** [0.00013 0.026]
*Random effects*		
Cluster variance	0.59 [0.20 1.69]	0.56 [0.17 1.84]
Number of clusters	400	400
Number of women	4,868	4868

Notes: *p < 0.05, ** *p* < 0.01, *** *p* < 0.001. This sample is also restricted to women who attended ANC at least once during pregnancy

There is no difference in the odds of a stillbirth for deliveries by skilled and unskilled providers when other factors are accounted for. However, the difference by type of delivery facility is still present; with 64 and 74 % lower odds of having a stillbirth for deliveries in a government health center/health posts and that in private facilities respectively, compared to deliveries in government hospitals and polyclinics. The odds of delivering a stillbirth is not significantly different for deliveries in government health centers or health posts compared to that in private facilities; and for deliveries at home compared to deliveries in government hospitals or polyclinics. Having some intervention during delivery is associated with about two times higher odds of having a stillbirth—likely due to selection, as women who have problem pregnancies are more likely both to have some medical intervention and a stillbirth.

Some variables related to health service utilization are omitted from the final multivariate models because of strong correlations between them. For example, delivery by a SBA, the type of delivery provider, delivery in a health facility, and type of delivery health facility are very strongly correlated (about 99 % of deliveries by SBAs occur in a health facility; and about 80 % of deliveries by doctors are in government hospitals or clinics). To examine each of these in multivariate analysis, separate models were run with only one or two of the delivery provider or facility variables included. In all cases, there was no significant difference for the binary variables—delivery by a SBA or not and delivery in a health facility or not—in the full multivariate models, but the difference by detailed type of delivery provider and type of facility were still present. When these two variables are included together only the difference by type of delivery facility is still present. A similar approach was used for the type of ANC provider and facility, none of which are significant in the final multivariate model.

Net of other factors, reporting a complication in the index pregnancy, a past stillbirth, a multiple gestation, and having a sister who died from pregnancy complications are associated with three to six times higher odds of delivering a stillbirth. Age and gravidity are both not significant in the final multivariate model (using both the continuous and categorical forms of the variable). Being never married is associated with about three times higher odds of having a stillbirth compared to currently married. A significant difference between the two multivariate models is seen in the effect of urban residence. Net of other factors including quality of ANC, urban residence is still associated with about two times higher odds of having a stillbirth. However, this difference is no longer significant when we include the delivery variables (the effect is also not significant when we exclude the intervention during delivery variable). Women living in the Eastern, Brong Ahafo, and Northern regions have over two higher odds of experiencing a stillbirth compared to women in the Greater Accra region.

### Additional analysis

#### Mediation and moderation analysis

The effects of education and wealth are not significant even without ANC quality in the model, hence do not warrant a formal mediation analysis. The rural/urban effect that is mediated by ANC quality is also not significant. For the question on whether the effect of ANC quality is mediated by the delivery place or provider, the change in the size of the coefficient for quality of care in the two logit models cannot be examined as the effect of quality of care mediated by the delivery variables, as part of the change is due to the rescaling of the logit model with the additional variables [31, 32]. Using the ‘khb’ rescaling method however shows that the ANC quality effect mediated by the type of provider alone is not significant, but the type of provider and facility and whether or not the woman had a medical intervention mediates about 14 % (*p* = 0.024) of the ANC quality effect; this suggests that a small amount of the effect of ANC quality on birth outcomes is through the use of facilities and providers with interventions that decrease the odds of having a stillbirth, but there is a much bigger direct effect. About 27 % (*p* = 0.020) of the urban effect is also mediated by these delivery variables. The mediated effects are calculated using the difference in the coefficients from the rescaled reduced and full models shown in Table 4. None of the interaction terms were significant; suggesting the effect of ANC quality is not conditional on the delivery provider or place, SES, or place of residence.

**Table 4:** Mediation by delivery care variables using the ‘khb’ rescaling method for regression on pregnancy outcomes

	*Rescaled reduced model*			*Full model*			*Difference (Full-reduced)*			*Proportion mediated by delivery variables*
Key Independent variables	coef.	SE	*p*-value	coef.	SE	*p*-value	coef.	SE	*p*-value	coef. of difference/reduced
Higher ANC quality	−0.61	0.27	0.02	−0.70	0.27	0.01	0.09	0.04	0.02	−0.14
Urban	0.80	0.37	0.03	0.58	0.37	0.12	0.21	0.09	0.02	0.27

Note: The rescaled reduced models exclude the delivery variables, but include their residuals. The full model includes the delivery variables (the same as the full model in Table 4)

#### Individual quality measures

To examine which of the individual components of the ANC quality measure may be contributing most to the differentials in birth outcomes, each of the individual variables used to create the ANC quality measure were regressed on the variables in the final models. These results showed that whether or not women were provided education during ANC on signs of pregnancy complication had the strongest effect, with women who were educated having about 50 % lower odds of a still birth than those not offered education (OR = 0.47 CI [0.27 0.79]).

#### Sensitivity analysis

To test whether the findings for ANC quality are dependent on the use of a particular cut-off point for high quality ANC, I estimated models in which ANC is included as a continuous measure (number of services received during ANC). In the multivariate models, each unit increase in the ANC quality score is associated with about 20 % decrease in the odds of delivering a stillbirth. An interaction term for ANC quality and number of ANC visits was also not significant. To test if the findings for women who attended ANC can be generalized to the whole population, I run the multilevel multivariate regression of birth outcomes using the full sample. Here women who did not attend ANC are given a quality score of zero and attending ANC is included as an indicator variable. Attending ANC at least once is not significantly associated with birth outcomes when other factors are accounted for, but attending four or more times is associated with lower odds of having a stillbirth as in the sample restricted to women who attended some ANC. The rest of the results are consistent with that for the sample of women who attended some ANC. In addition, I run weighted and unweighted single level logistic regressions on stillbirths using the restricted and full samples. These analyses all produce consistent results, suggesting the weights not used in the multilevel multivariate analysis do not significantly affect the findings.

## Discussion

A key question in this study is whether antenatal care (ANC) quality has an effect on women’s birth outcomes, net of other factors. The results show that higher quality ANC decreases the odds of having a stillbirth by almost half—after accounting for other factors. A small proportion of the ANC quality effect is through the use of delivery providers and facilities with interventions that may decrease the odds of delivering a stillbirth, but there is a significant direct effect of ANC quality on stillbirths, net of the delivery provider and facility. No prior study in Ghana has examined the effect of ANC quality on birth outcomes using a nationally representative sample of women. A few studies in different parts of the country have looked at certain components of ANC, but found no significant effects in multivariate analysis—potentially due to how the models were specified.

For example, a study based on a survey of women presenting for antenatal care at a health facility in the Ashanti region, found that women who did not receive malaria prophylaxis during ANC had higher odds of delivering a stillbirth in unadjusted models, but this association was not significant in multivariate models. The study, however, also had biological markers including laboratory diagnoses of malaria and folate and hemoglobin concentrations—potential mediators for the effects of receiving a malaria prophylaxis—and found higher odds of having a stillbirth with low folate, anemia, and malaria infection [19]. Another facility based study examined the effects of some components of ANC, including screening for anemia and helminthes, tetanus vaccination, and nutritional supplements on adverse birth outcomes, but none of these factors was significant in their multivariate models, potentially due to multicollinearity, as the ANC content variables, which are usually correlated, were all entered as separate variables in the multivariate model [34].

Evidence elsewhere show that antenatal interventions such as serologic screening for syphilis, iron supplementation, malaria treatment and prophylaxis, diagnoses and treatment of asymptomatic bacteriuria, blood pressure monitoring, anti-tetanus immunization, and prevention of mother-to-child transmission of HIV can improve fetal outcomes [35–38]. This study provides additional evidence for the role of receiving the recommended antenatal interventions in reducing stillbirths. If every woman who comes into contact with the health system during pregnancy receives the basic package of ANC services, it could substantially reduce the number of stillbirths in the country. Butta et al. (2011) project that a basic package of antenatal interventions including periconception folic acid supplementation or fortification, prevention of malaria, and improved detection and management of syphilis during pregnancy; and basic and comprehensive emergency obstetric care could avert up to 45 % of stillbirths [9].

The finding of this study on the individual components of the ANC quality measure also suggests that health providers need to go beyond taking blood pressures and samples to educating women during antenatal care. Women are less likely to report being educated on danger signs of pregnancy than being offered the other services (about a third of women reported not being educated on pregnancy complications, compared to less than 12 % for most of the other services (Additional file 2). The reason for this may be that it is much faster to take a blood pressure or a blood sample than to teach a woman the danger signs of pregnancy and what to do in the event of a complication. Providers may therefore skip this time consuming component of ANC to enable them get through the long queue of women waiting to see them. The finding here however suggests that taking time to talk to women during ANC could reduce stillbirths significantly. Providers need to be trained and motivated to educate and counsel women during ANC if women are to reap the full benefits of ANC.

The mediation analysis also suggests that good quality ANC may improve birth outcomes by promoting deliveries in health facilities where complications can be better managed. Net of other factors, deliveries in government hospitals or polyclinics are, however, associated with higher odds of delivering a stillbirth than deliveries in private facilities and lower tiered health facilities. Similar findings have been obtained elsewhere and this is primarily due to selection—not because delivery in health facilities or hospitals leads to poor outcomes. [17]. To the contrary, women at risk of stillbirths are more likely to deliver in health facilities, and even more likely to deliver in the higher tiered facilities, where they are usually referred to. Nonetheless, if we assume that skilled delivery should improve outcomes even for women with complications (which is the expectation for skilled attendance), then the findings raise two questions: (1) Are women with complications presenting to higher tiered facilities so late that not much can be done for their babies and potentially themselves? (2) Are health facilities not doing enough for these women? These are questions that cannot be answered based on the current analysis, but from other evidence, the answer is likely yes to both for a number of reasons.

First, poor quality ANC and delays in the decision to seek and reach skilled delivery care result in many women presenting very late at health facilities. For example, some ANC providers may not follow up on an initial blood test for anemia or sickle cell disease—risk factors for stillbirths—until a woman has developed severe anemia or sickle cell crises with a stillbirth; at which stage she is referred to a higher level facility, when it is too late to save the fetus. Women may also present at health facilities for delivery only after many hours of failed delivery at home, at which time health providers may not be able to do much to save the fetus. These delays suggest the broad indicator “SBA use during delivery,” which is frequently employed in studies of maternal health, may be misleading because we rarely know when women decide to seek skilled attendance. With a high proportion of late presentations, maternal health outcome indicators will continue to lag behind the crude skilled birth coverage indicators. A useful question for the large national health surveys will be when prior to or during labor women decide to seek the assistance of a SBA.

Second, the poor referral system increases the chance that the fetus, even if alive at referral from a lower level facility, will be dead by the time a woman reaches the referral facility, especially if fetal distress is a complication. In a recent assessment of Emergency Obstetric and Neonatal Care (EmONC) in Ghana, 46 % of facilities reported not making any transportation arrangements for clients referred to higher facilities [39]. This finding implies that the burden of finding and paying for appropriate transportation is borne entirely by the woman and her family, which further increases delays to reaching a more highly skilled facility. Some of these factors explain the higher frequency of stillbirths in government hospitals and polyclinics, which are the referral points for lower tiered government health facilities and private facilities.

Third, many health facilities in Ghana including referral facilities are understaffed, underequipped, and lack basic drugs and supplies needed to avert maternal, fetal, and early neonatal deaths; thus are not able to do enough for women presenting to them. Many maternal and fetal deaths that occur in health facilities can be linked to delays in receiving timely adequate care after arrival [40, 41]. The doctor-to-population and midwife-to-population ratios of about 1 to 10,032 and 1 to 1,478, respectively, in Ghana fall far below the minimum threshold of 23 health workers per 10,000 population needed to deliver essential maternal and child health services; with substantial shortage of trained surgeons who can perform obstetrical procedures (e.g., cesarean sections) at first level-referral facilities [42–44]. Furthermore, very few of the facilities that should provide basic EmONC are able to effectively do so, and deficits in the management of labor including inadequate use of partographs and non-use of recommended treatments are common [39, 45, 46]. These inadequacies lead to instances where women are admitted in labor with live fetuses and deliver stillbirths after several hours of labor, because of inadequate monitoring of labor to identify problems and initiative appropriate interventions. Worse still, in some cases, fetal complications requiring immediate delivery are diagnosed, but caesarian section is delayed because of multiple emergency caesarian sections and only one operating theatre, one doctor, and/or one anesthetist on duty. Thus, although there are cases of health provider negligence and incompetence, many stillbirths are due to systemic problems.

The other factors which increase the risk of a stillbirth—pregnancy complications, multiple gestation, and a past stillbirth—are well known risk factors [2, 6, 11, 24, 47]. It is unclear what may be accounting for the strong significant association between having a sister who died from pregnancy complications and having a stillbirth. Possible reasons include the familial component of some risk factors for both maternal deaths and stillbirths, like hypertension and diabetes. It may also be due to poor access to good quality health care by women with sisters who may have been affected by similar contextual factors. In addition, since a woman has to have a sister who was pregnant for her to die from pregnancy complications, women reporting sisters who died from pregnancy complications may over represent women from large families, who may be more likely to have large families themselves—a risk factor for stillbirths. The number of women in this sample with sisters who died from pregnancy complications is small; but the consistent strong effect in the multivariate models suggest this association is likely not spurious. More studies are needed to understand the underlying process, but this finding adds to the evidence on the strong relationship between risk factors for adverse maternal and fetal outcomes and the utility of examining stillbirths as a measure of adequacy of maternal care, including for the role of contextual effects.

Studies in high income countries show socioeconomic differentials in stillbirths, but these differentials are more common for intrapartum stillbirths than antepartum stillbirths [2, 11, 48–50]. Few studies have however explicitly examined socioeconomic differentials in low income countries [17]. Like this study, none of the studies in Ghana found an effect of education. None also found an effect of wealth, except for one study. This study found that women in the poorest wealth groups had the highest risk for intrapartum stillbirths, but there was no association between antepartum stillbirths and wealth [17]. The non-significant effect of SES in our study and the other studies in Ghana may therefore be because we were unable to distinguish between antepartum and intrapartum stillbirths. The stronger effect of wealth on intrapartum than antepartum stillbirths is said to be because antepartum stillbirths have more multifactorial causes that may have a genetic component and may be unrelated to use of health services [17, 48–52]. Nonetheless, evidence suggests better access to good quality antenatal and delivery care can decrease both antepartum and intrapartum stillbirths [8, 9]. The other potential reason for the non-significant effects of SES is the opposite effects of their intervening factors, which may suppress their effects. For example, higher SES may be associated with older age at first birth, which increases the risk of having a complication that may result in a stillbirth. On the other hand, higher SES women are more likely to use and receive higher quality care, which decreases the risk of having a stillbirth.

The association between place of residence and birth outcomes is another finding worth noting. Rural areas are said to account for a larger proportion of stillbirths globally, and especially in SSA [1]. The findings from the GMHS however shows that while rural areas have a larger absolute number of stillbirths (potentially because of higher fertility), the proportion of all births that result in a stillbirth is higher in urban areas than rural areas [13]. From other analysis, we know women in urban areas are more likely to use skilled providers and health facilities for delivery. That the urban effect is no longer significant when the delivery provider and place of delivery are added to the model suggest that women in urban areas may have higher biological or other risk factor for having a stillbirth, but this risk is reduced by the type of care they receive during delivery—i.e., if deliveries in health facilities were not as high as they are in urban areas, the risk of stillbirths will have been much higher in urban areas. The regional differences are more difficult to explain, although accessibility to health facilities and differential quality of delivery care are potential factors. These regional differences present an area for further research.

### Limitations and strengths

This analysis has a number of limitations. First, the study is based on cross-sectional data, hence has the limits on causal inference inherent in any cross-sectional analysis. The data are also based on self-report, thus subject to recall and social desirability bias. Furthermore, the numbers of stillbirths by subgroups are small because of the small number of stillbirths in the sample. Stillbirth rates from surveys are said to be underestimated due to misreporting, and the stillbirth rate from this analysis, which looks at only the last birth (because the quality of ANC questions were only asked of this birth) of about 17 per 1000 pregnancies is lower than that for all births in the preceding five years (21 per 1000 births) for the same data [1, 13]. This is because all live births in the preceding five years will include multiple births for some women especially those with short interpregnancy intervals, who are also more likely to have stillbirths [13]. This should however not significantly affect the results, as the purpose of the analysis is not to provide estimates of the stillbirth rate, but to examine associations; and controlling for past stillbirths helps account for other pregnancies in the survey period that may have resulted in stillbirths.

The measure of quality of ANC is also limited in discriminating between different levels of quality of care and does not adequately capture all the dimensions of quality. The questions in the GMHS are useful for evaluating whether or not women are receiving the essential ANC services, but they do provide adequate information to distinguish between receipt of basic services and more advanced care. They are also insufficient to determine if women who had various screening tests were adequately followed up and appropriately managed [21]. In addition, other components of care such as those related to prevention of malaria were not assessed in the survey, thus not included in this index. The findings of the effect of quality despite these limitations suggest the magnitude of the effect of ANC quality is potentially higher than shown in this study.

The other limitations relates to the lack of data on some variables that are related to the birth outcomes and the dependent variables. The first of these is the lack of data on whether the stillbirth was antepartum or intrapartum. The proportion of antepartum and intrapartum stillbirths from other studies are about 40 to 60 % and 15 to 40 % respectively in different settings [2]. A study in the Brong Ahafo region in Ghana found about 53 % of stillbirths were antepartum and 38 % intrapartum (9 % unclassified from missing data) [17]. Thus, this sample likely includes a good mix of antepartum and intrapartum stillbirths. The findings regarding the effect of ANC utilization and quality are, however, more consistent with findings for antepartum stillbirths, suggesting the sample may include a larger proportion of antepartum stillbirths. Data on pregnancy duration is only available for stillbirths, thus pregnancy duration is not examined as a predictor in the analysis. This should however not be a major problem, because prematurity as a risk factor for stillbirths [16, 24], is more of an intervening factor—there are usually other factors antecedent to prematurity that indirectly affect the occurrence of stillbirths; and accounting for these antecedent factors may be more important. There is also no specific data on maternal conditions including chronic diseases, body weight, malaria, and anemia during pregnancy. However, the effects of most of these factors are likely captured by other variables like report of a complication or an intervention during delivery. Other risk factors missing from this data are use of alcohol and smoking during pregnancy. Studies in Ghana have however suggested these are very rare among pregnant women [17, 19].

The omission of variables related to the focal independent and dependent variables from the analysis increases omitted variable bias hence problems of endogeneity and unobserved heterogeneity [25, 31]. The other source of endogeneity—simultaneity or reverse causation may be less of a problem for the focal relationship as it is highly unlikely that the birth outcome will cause the quality of ANC for the index pregnancy. The reverse is more plausible, which increases confidence in causal inference based on the temporal ordering of the events. However, simultaneity is a problem for the place and type of delivery attendant as women may seek care only after they realize they have a problem, as discussed on the selection of high-risk women to deliver in higher-level facilities and by skilled providers. The findings for the delivery variables should therefore be interpreted with caution.

The study has several strengths. First, it addresses a gap in the maternal health literature: the dearth of quantitative studies examining the relationship between process and outcome measures of quality of maternal care. Second, it uses a nationally representative sample of women who had a birth in the five years preceding the survey, hence has high generalizability. Unlike analysis based on the usual DHS, which will include only women with a live birth (the group that are asked the maternal health questions), the GMHS includes all women with a birth (live or otherwise) in the preceding five years, which has made this analysis possible. To my knowledge, this is the first study in Ghana to examine the predictors of stillbirths based on national data. Restricting the sample to women that had at least one ANC visit, though necessary for the analysis on quality of care, may reduce the generalizability of the findings. But this represents over 95 % of women in Ghana. Understanding the determinants of birth outcomes in this population is important because this is a potentially more accessible population, easier to target for interventions. Furthermore, the sensitivity analysis shows that the findings are not significantly different from that of the full sample. The analysis also uses rigorous methods and the sensitivity analysis shows the findings are robust.

## Conclusions

This study finds that high quality ANC decreases the odds of delivering a stillbirth net of other factors. High quality ANC can improve birth outcomes in two ways: directly through preventative measures and indirectly through promoting deliveries in health facilities where complications can be better managed. There has been a big emphasis on improving coverage for maternal health services; with relatively less emphasis on the quality of care women receive. More efforts are needed to improve quality of maternal health care. ANC is an opportunity to identify women with risk factors for stillbirths and start appropriate follow-up care. All women should also be educated on danger signs of pregnancy during ANC and on what to do in the event of complications. Improving access to maternal health services is obviously very important, but use of services will not result in the desired outcomes if it is not associated with receipt of good quality care. Other analysis showed women who receive ANC from the health centers and other lower level facilities are more likely to receive low quality ANC [21]. Improving ANC quality in these lower level facilities will reduce the number of women presenting in labor with unsalvageable conditions. This study did not have the required data to examine the role of quality of delivery care. But there is evidence elsewhere to suggest poor quality delivery care is also contributing to high intrapartum stillbirths and maternal deaths [7, 11, 53]. A call for greater efforts to improve quality of maternal health services from antenatal through delivery to postnatal care in Ghana and the rest of SSA is therefore not out of place.

## Abbreviations

ANC, Antenatal Care; DHS, Demographic and Health Surveys; EmONC, Emergency Obstetric and Neonatal Care; GMHS, Ghana Maternal Health Survey; SBA, Skilled Birth Attendant; SES, Socioeconomic Status; SSA, Sub-Saharan Africa; WHO, World Health Organization

## Additional files

Additional file 1: Distribution of variables for full sample, GMHS (XLSX 50 kb) Additional file 2: Distribution of individual components of the ANC quality measure, GMHS (XLSX 9 kb)
